# Investigating Managers’ Fine-Grained Evaluation Processes in Organizations: Exploring Two Dual-Process Perspectives

**DOI:** 10.3389/fnins.2021.649941

**Published:** 2021-09-01

**Authors:** Haoye Sun, Willem J. M. I. Verbeke, Frank Belschak, Jan van Strien, Lei Wang

**Affiliations:** ^1^School of Management, Zhejiang University, Hangzhou, China; ^2^University of Hamburg, Hamburg, Germany; ^3^Erasmus University Rotterdam, Rotterdam, Netherlands; ^4^Faculty of Economics and Business, University of Amsterdam, Amsterdam, Netherlands; ^5^School of Social and Behavioural Sciences, Erasmus University Rotterdam, Rotterdam, Netherlands; ^6^Neuromanagement Lab, Zhejiang University, Hangzhou, China

**Keywords:** economic context, social network context, dual-process theory, event-related potentials, stereotype content model

## Abstract

The dual-process theory is a significant theory in both organizational theory and social psychology and two conjectures about this theory are considered in this manuscript; the default-interventionist vs. parallel-competitive account. Our research goal is to empirically investigate these two views. In concrete terms, by using event-related potentials (ERPs), we seek to study the fine-grained brain processes and self-reported feelings involved in managers’ evaluations of target employees within an economic context (firing employees) vs. a social network context (excluding employees). Using the stereotype content model categories, each target employee has high (or low) warmth and high (or low) levels of competence. In the fine-grained ERP analysis of the brain process, we focus on three time windows of interest: novelty detection (N2) and goal violation detection (N400) at the unconscious level, and we then evaluate conscious emotional arousal (late positive potential, LPP). Finally, we focus on the self-reported feelings when having to fire or exclude target employees. As goal pursuit theory predicts, the brain dynamics and self-reported measures differ widely across the two organizational contexts; in concrete terms, processes at a later stage overrule early stages depending on the context. This implies that the data bespeaks more for the parallel-competitive account than the default-interventionist account. We discuss the implications of these findings for research in management and management practice.

## Introduction

How do managers make managerial decisions about different issues in their organization, such as whether they have to fire employees from the firm or exclude employees from their social network? We focus on the evaluation or appraisal that managers need to undertake before they decide on either firing employees for economic reasons or excluding employees from their social network to retain a cohesive and productive social network. Managers, in general, make both economic and social (network) decisions and these interpersonal decisions reflect two vital communal vs. agentic goals people in general seek to attain (Bakan, 1966; Bass and Stogdill, 1990). Firstly, a manager’s goal is to keep an eye on the financial health of the firm, and in some cases, the least productive employees might need to be fired to keep the firm financially healthy. Secondly, managers should strive to surround themselves with a cohesive social network of employees where they experience psychological safety, but if a skirmish occurs within their social network, e.g., due to the harmful attitude of one or more people, managers will exclude these people from their social network, which allows the productivity of network members to increase again. This study focuses on these two fundamental managerial decision processes.

In the progression of organizational and social neuroscience, researchers have become interested in the brain processes underlying managerial decision-making and information processing, and insights gained from this research might lead to a better understanding of how managers make these decisions (Becker et al., 2011; Senior et al., 2011). One of the reasons for taking an interest in this research topic is that some research has shown, when managers make evaluations, they are already very involved in their evaluation process in early and unconscious phases, and self-reports occurring at the conscious level cannot grasp these early phases of the evaluation (Becker et al., 2011). A typical example is discrimination based on social stereotypes (Amodio, 2014), and in both the management and social psychology literature, it is known that discriminating against employees involves a dual decision processing modus (Hodgkinson and Sadler-Smith, 2018). The neural dynamics affecting the dual-decision processing, however, is currently under discussion and two views have been proposed: the default-interventionist account and the parallel-competitive account (for an overview see Hodgkinson and Sadler-Smith, 2018). The main aim of this manuscript is to investigate whether the data gathered using Event-Related Potentials (ERP)-based research and self-reported feelings (outcomes) that follow the evaluation in a laboratory environment, provide evidence for one or both views. In order to study these two dual processes, we categorize target employees according to the stereotype content model (SCM). Note that these SCM categories, affecting neural and psychological processes involved in decision-making outcomes, have already been studied in general (Quadflieg et al., 2009). So too, how it might be moderated by social contexts, such as evaluations involving target people differing in race (Amodio, 2014), being part of less or more powerful groups (Cuddy et al., 2008), as well as in organizational contexts (Cuddy et al., 2011). To our knowledge, however, we do not know of any study that focuses on how specific organizational contexts affect whether aspects in the SMC categories become more or less relevant during the neural and psychological processes involved in decision-making. More specifically, by using such a laboratory research design, we seek to uncover whether there is evidence of the two different views in the dual-process theory.

The manuscript is structured in the following manner. Firstly, we explain the two dual-process theories investigated in this manuscript: the default-interventionist and the parallel-competitive account. We also explain how ERP-based research and psychology-based research in two different organizational contexts are simulated in a laboratory context. Here, the decisions made by managers about firing (economic context) or excluding target employees (social network context) who have been categorized according to the stereotype content model (SCM), might shed light on what those theories might mean for managerial decision-making. Then we explain what ERP-based research can reveal about the neural and psychological dynamics of managerial evaluation processes, and make hypotheses about how aspects of the SCM elicit different ERP amplitudes during three continuous time windows in an economic and social network context. Next, we describe how aspects of the SCM might affect differences in the intensity of self-reported comfortable/uncomfortable feelings when having to make firing/exclusion decisions in these two differing contexts. Then, we describe the methods, report the results, and discuss how this study is relevant for managerial research as well as for limitations.

## Theories

### Dual-Process Theories

Recently, Hodgkinson and Sadler-Smith (2018) considered two perspectives or accounts in the dual-process theories of managerial decision-making: one is the default-interventionist account (and originates from e.g., Kahneman and Frederick, 2002) and the other is the parallel-competitive account (and originates from e.g., Lieberman, 2000). Firstly, the default-interventionist account of dual-process models argues that Type 1 processes, evolutionarily reflect older brain processes and allow a person who makes evaluations to save memory space needed for Type 2 processes. These Type 1 processes, however, are the drivers of the evaluation process and most importantly, Type 2 processes cannot override Type 1 processes. This phenomenon is known to provoke bias in decision-making. The second theory, considers Type 1 decision processes as more unconscious, inflexible, holistic, automatic and evolutionarily old, while Type 2 decision processes are considered to be explicit, flexible, abstract and logical and both types of processes, however, compete during the process model, allowing later stages to override the earlier processes (Evans, 2008; Frith and Frith, 2008).

Both perspectives have implications for managers. For instance, human resource managers in European firms might be more likely to refuse hiring job candidates with dark-colored skin as opposed to white candidates, and this real discrimination reflects their true preferences or attitudes. However, when asked later whether they genuinely discriminate against colored job applicants, they would answer in a socially desirable way, indicating that they always act professionally and treat every job applicant equally no matter the color of the person’s skin. This phenomenon is sometimes called the “two minds in one brain perspective” (Evans, 2003). It implies that the managers’ desirable answers are only rationalizations of decisions already made at an early stage in the decision-making process, which is the case for the default-interventionist account of occurrences during the early decision stages (Type 1) that biases or guides decisions at later stages (Type 2) (Hodgkinson and Sadler-Smith, 2018). The parallel-competitive account challenges this assumption. It assumes as a fact that Type 1 and Type 2 processes operate in competition with one another and “the process that completes first, cues a response,” which may then be inhibited by an alternative, less rapidly cued process. This leaves room to avoid biases in decision-making (Hodgkinson and Sadler-Smith, 2018). Hence, managers’ decisions are not always rationalizations of decisions already made at an early stage in the decision-making process, but these can be flexibly achieved by managers depending on the goal of the decision making or social context.

### Stereotype Content Model

The stereotype content model was first proposed by Fiske et al. (2002) to investigate people’s emotional responses to different stereotype categories. SCM defines a matrix of 2 (low/high warmth dimension) × 2 (low/high competence dimension) to present four broad stereotype categories: Groups high on both warmth and competence, groups low on both warmth and competence, and mixed quadrants (high on one of the two dimensions) (Harris and Fiske, 2006). The four combinations of high vs. low warmth and competence judgments create four unique emotional responses. More specifically, the two extreme groups elicit people’s admiration (all-good group) and disgust (all-bad groups). As for the two ambivalent groups, the “competent but cold” group elicits envy, while the “warm but incompetent” group elicit pity.

## Hypotheses

Following the evaluation process, we investigated three different ERP components before analyzing the self-reported feelings. In the time windows of interest referred to in this manuscript, the ERPs have been widely used in previous stereotype-related studies (White et al., 2009; Hehman et al., 2013; Hilgard et al., 2014). The first phase of the evaluation process involves the detection of novel stimuli. As the goal of the evaluation process is to fire or exclude a “target employee” from one’s social network in this time window, the brain (N2 component) only prepares a person to engage in a new task (which is “to fire or exclude the specific employee shown in a picture”). The N2 component is a negative deflection, which is typically evoked in approximately 200–300 ms over frontal-central brain regions (Folstein and Van Petten, 2008). N2 activity can be seen as a reflection of early attentional processing (Endendijk et al., 2019) and unconscious information processing (Caldwell and Riby, 2007). N2 has been interpreted as indexing stimulus unfamiliarity, attentional shift or the detection of novelty (Daffner et al., 2000; Nittono et al., 2007).

The second ERP component in the second time window implicates whether a social stimulus evokes goal violation or goal incongruency. In our task, this component reflects whether the social stimuli match the goals that a manager seeks to achieve and is called the N400. The N400 component is a negative deflection that appears approximately 400 ms after the stimuli presentation (Kutas and Hillyard, 1980, 1984). N400 reflects early automatic unconscious processes and is widely used in semantic and non-semantic contexts, as an index of a cognitive mismatch (Kutas and Federmeier, 2011; Huang et al., 2014; Rohaut et al., 2015). In particular, and most relevant for this study, a larger N400 amplitude was also found to appear in action-goal violation conditions (Reid and Striano, 2008; Balconi and Caldiroli, 2011). For example, when the goal is to drink (bringing a cup to the mouth), an image of an “eye” elicited a more negative N400 amplitude than an image of a “mouth” (Amoruso et al., 2013). In applying these findings to managerial contexts, the conjecture is that when the target employees cannot fit into the managers’ goals, a more pronounced N400 amplitude will be observed.

The next stage of the evaluation process is the late positive potential (LPP) and measures the degree to which a stimulus is emotionally arousing and occurs at the conscious level. The LPP component is a sustained, positive component that occurs between 300 and 1,200 ms after stimuli presentation, reflecting the late, elaborate, and conscious cognitive process of (explicit) evaluative categorization (Ito and Cacioppo, 2000; Schupp et al., 2000; Wang et al., 2016). It is widely recognized that an enhanced LPP amplitude represents more emotionally salient arousal (both positive and negative) and more motivated and sustained attention to emotional stimuli (Hajcak et al., 2012; Larson et al., 2012). Similarly, the LPP amplitude is found to increase by emotionally arousing targets, but not indifferent targets (Czekóová et al., 2015). When participants are indifferent to targets, they tend to perceive the target as neutral and thus evoke lower emotional arousal (Delaney-Busch et al., 2016). In the managerial task, extreme employees might evoke greater emotional arousal compared to ambivalent employees, because managers tend to have mixed emotions (like-dislike offset) toward ambivalent employees, but unmixed emotions toward all-good (like) and all-bad (dislike) employees.

With regard to the self-reported feeling measure, an important component of the SCM is the feelings that are evoked when a person is confronted with people belonging to different SCM categories (Fiske et al., 2007). For instance, people high in competence but low in warmth evoke envy, while people high in warmth and competence evoke admiration (Cuddy et al., 2007). In our case, the feelings of comfort/discomfort that occur in making decisions about firing or socially excluding a target employee are reported.

Having provided these explanations of the consecutive brain processes, we are now better able to hypothesize the evaluation processes in different contexts. We argue that the conscious goals of the managerial evaluation/decision, which are activated by the contexts or tasks, are the drivers of the brain processes. These goals imply that the manager makes some SCM categories more relevant or salient than others, and are thus related or associated with differences in ERP amplitudes in the corresponding time windows of interest and differences in intensity of reported comfortable/uncomfortable feelings, within the two contexts. Hence, the manager’s decision or evaluation is not necessarily determined or controlled by the earlier phases in the decision-making. Therefore, the parallel-competitive model is more likely to be a better description of managerial decision-making than the default-interventionist model. We consider this logic as the overall conjecture to be tested in the manuscript. Detailed hypotheses regarding different evaluation stages are proposed as follows:

Note that, although we measure the N2 component, we do not expect it to be affected by aspects of the SCM, as it reflects novelty detection (which means being confronted with a new or specific person to be fired or socially excluded).

In an economic context, the goal of the managers is to keep the company financially healthy, and thus, they think about employees in terms of financial ratios and performance indicators. Hence, this goal ensures that the performance (competence) dimension of the SCM is the most relevant driver of managers’ evaluation processes about target employees. This means that managers are most likely to eliminate low-performing employees rather than high-performing employees. Accordingly, we assume that brain activity of the N400 component will elicit a higher amplitude when confronted with employees exhibiting low performance than with employees exhibiting high performance; in the first case, low-performing employees violate the managers’ economic goals, and the better-performing employees do not violate but rather conform to the managers’ economic goals. We also assume that the managers will remain insensitive to the warmth of the employee, as it is not relevant for achieving their financial goals. Thus, the warmth dimension of the SCM will not elicit differences in the N400 amplitude in the corresponding time window, and neither will it affect differences in other ERP components in further time periods. By the same token, the amplitude of the LPP will be high when managers encounter an employee exhibiting either high or low performance because, in the first instance, the manager is emotionally aroused; experiencing excitement about the performance, but in the latter instance, the manager is emotionally aroused; experiencing anger or disappointment about the performance. Finally, in asking the degree to which the managers feel comfortable/uncomfortable when firing the employee, it is obvious — given the economic goals — that they will feel uncomfortable firing the better-performing employees, but less uncomfortable firing the lower-performing employees.

Where the evaluation processes in the social network are concerned, we follow the insights provided by Pfeffer (1989) that, although competence matters to managers as indicated in the economic context, managers also seek to build their power base (i.e., their social network) by hiring an employee who is much like themselves (Mcpherson et al., 2001; Kilduff and Brass, 2010), as similarity has been found to increase social warmth (Byrne, 1971; Welkowitz and Kuc, 1973). Similarly, being able to operate in social networks requires social skills, which means being able to show warmth toward employees (Kilduff et al., 2006). Note, however, that within a social network, competence also plays an important role, as the aim is to build productive groups/teams in organizations (Burt, 2000; Brass, 2011). An employee in social networks, who is not cooperative or does not add value, will more likely be excluded (Watanabe et al., 2017). So, as Brass (2011) mentioned, individual characteristics (e.g., social skills) (Mehra et al., 2001) and individual activities (Zhou et al., 2009) both matter in social networks. Therefore, we expect managers to focus most on an employee in the all-good group and this category will evoke a lower N400 amplitude from managers than for employees in other categories. Employees in the two ambivalent categories and the all-bad category, more or less violate managers’ goals in the social network. Employees with high levels of warmth but low levels of performance do not contribute to the network. Similarly, the low warmth but high-performing groups are not perceived as part of the network despite their financial contribution. In addition, all-bad (low warmth and low-performing) employees are neither part of the network, nor do they contribute to the network. Thus, we expect ambivalent and all-bad employees to evoke a larger N400 amplitude from managers due to the violation of managers’ needs for both performance and social skills in the social network. Next, employees with high levels of warmth and competence will emotionally arouse managers and elicit a larger LPP amplitude, than employees with low levels of warmth and competence, but so will employees in the low performance and low warmth group, as they irritate the manager with their poor social skills and performance. Regarding the ambivalent groups, we cannot make strong hypotheses; an employee with a low level of warmth but a high level of performance might not evoke much arousal (hence a low amplitude at the LPP time window), as they are considered to be competent jerks who do not seek to be included in the social network and managers might feel indifferent toward them (Casciaro and Lobo, 2005). Finally, we expect that managers will report more uncomfortable feelings when asked to exclude the group with high levels of warmth and high levels of competence, as they are the biggest drivers of cohesion and productivity. All other groups will evoke less discomfort when being excluded from the social network.

## Materials and Methods

### Participants

Permission for conducting this study was granted by the Ethics Review Board of the university. Since the study has a management focus, only students with related backgrounds were selected to participate in this experiment. A total of 60 business school students (35 males and 25 females) signed up voluntarily to take part in our experiment for which they would obtain grade credits. All the participants had normal or corrected-to-normal vision and reported no history of psychiatric problems. They were informed about their right to stop participation in the experiment at any time, if they wanted to. Every participant was assigned to a within-subject design. Note that our design is relatively complex in comparison to other EEG studies as it reflects specific organizational contexts; therefore, seven of the participants were tested in a pilot study to ensure that they could fully understand the design. Eight of the participants were excluded because of excessive artifacts in their brain signals (>20% trials were rejected as artifacts), leaving 45 valid participants for data analysis (mean age = 23.71 years, SD = 2.48, 21 females). The effect of gender did not play a role in further analysis.

### Stimuli

Referring to the stimuli in previous studies (Cikara et al., 2010; Harris and Fiske, 2010), 200 portrait images of target employees were acquired from the internet based on the examples given in previous literature (e.g., elderly people tend to be judged as having low competence but high warmth), including 50 possible stimuli in each category (high warmth, high competence; low warmth, low competence; high warmth, low competence; low warmth, high competence) in our pretest. A total of 180 participants who were recruited using Amazon Mechanical Turk (MTurk), were instructed to imagine that they are the managers of a firm and that they need to evaluate the warmth and competence of all 200 portraits of 200 employees using a 7-point scale (1 = low level, 7 = high level). Twenty-five outstanding portraits of target employees fit the descriptions of every category and most were put into the corresponding category pool. However, fewer portraits of employees were selected in the high warmth but low competence category, based on evaluations by 180 participants. Another 50 possible high warmth, low competence portraits were then downloaded from the internet and evaluated by another group of MTurkers (*N* = 529) who had not participated in the first evaluation round.

**FIGURE 1:**
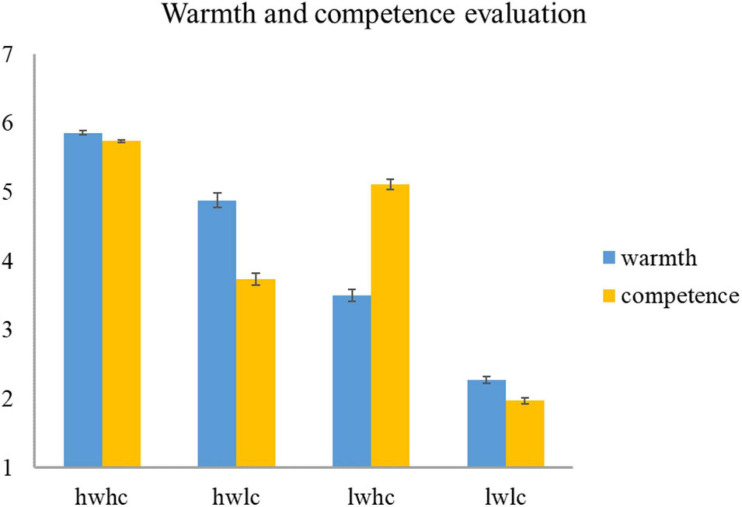
Descriptive statistics for warmth and competence in four categories (hw, high warmth; lw, low warmth; hc, high competence; lc, low competence).

In the end, 100 portraits of employees – equally distributed into four categories – were selected from the ratings provided during the two evaluation rounds. Figure 1 shows the mean value and average standard error of the 25 selected pictures in every category.

### Design of the Experiment

**FIGURE 2:**
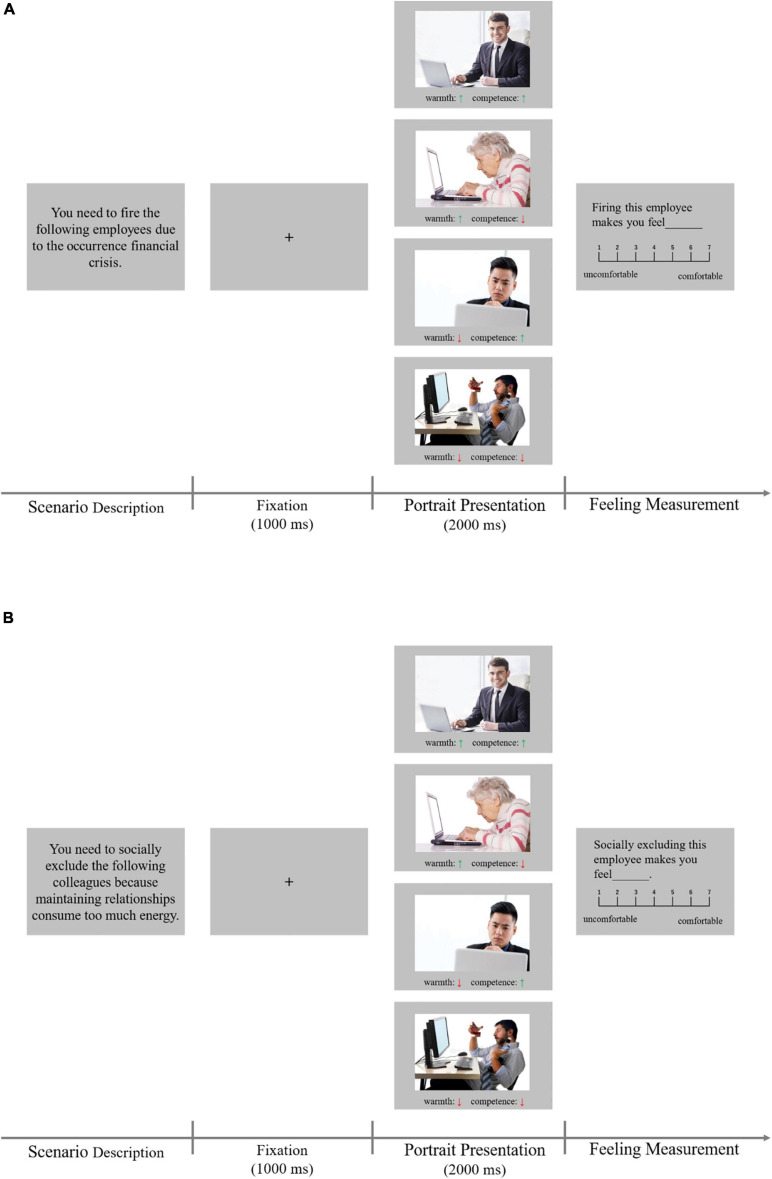
Timeline of the experimental design. **(A)** Economic context. **(B)** Social network context.

The whole experiment consisted of two blocks: the economic and social network contexts. In the economic context, participants were asked to imagine that they are the managers of a firm who faced significant economic difficulties endangering the survival of the organization; this meant employees needed to be fired. Their purpose for making this decision was to clearly ensure the firm’s financial development and growth. In the social network context, participants were asked to imagine that they are the managers of a firm, and in their social network, they would face some issues that would require some employees to be excluded from the network; otherwise, the network would not remain viable or productive. Their purpose for this task was clear: They had to keep a comfortable and harmonious social relationship in their social network. These two kinds of manipulations; firing an employee and socially excluding an employee, have been studied in previous literature but in a discrimination context (Hebl et al., 2002; Puhl and King, 2013). The sequences of the two blocks were counterbalanced. In each block, 25 scenarios were presented. Afterward, portraits of employees in four SCM categories appeared one by one on the screen, resulting in 25 trials per category per context. The combination of four employees under one scenario was maintained in the two contexts, excluding the impact of uninteresting stimulus combinations. Participants were then asked to evaluate their feelings about firing, or as the case may be, socially excluding the employee in each picture. Figure 2 illustrates the timeliness of the experimental design.

In each trial, an exclamation mark was presented for 500 ms, after which the scenario was described, explaining the reasons why participants needed to either fire or socially exclude employees. They were asked to either fire (or not) or socially exclude (or not) the employee being presented. After they had finished reading the contents, they could press the space bar to continue, and no time limitation was set for reading.

Afterward, four portraits of employees in four categories were presented one by one in a random order. The presentation of every target employee started with a 1,000 ms fixation, followed by the portrait of the employee in the middle of the screen. Note that to avoid the halo effect of the two dimensions (Yzerbyt et al., 2008; Kervyn et al., 2009) and different perceptions of every picture, two horizontal-alignment arrows were presented below the portrait, representing a high or low level of warmth and competence, respectively, guiding them to evaluate an employee with respect to the warmth and competence dimensions. After the employee had been shown for 2 s, self-reported feelings were measured. This was done particularly to avoid the tendency to conceal genuine attitudes (at least, not expressing them), which is widely observed in previous literature (Quillian, 2006; Martinez et al., 2016). We did not ask participants to pick a target employee to fire or to socially exclude; instead, participants were asked to insert a rating: “Firing or socially excluding this employee makes you feel ______” by typing the corresponding number on the 7-point scale (1 = uncomfortable, 7 = comfortable). No time limitation was set for the feeling evaluation stage.

### EEG Recordings and Analysis

An EEG signal was recorded during the entire experimental process from 34 Ag/AgCI electrodes (standard 10–20 layout with two extra electrodes at FCz and CPz) placed on a cap connected to a BioSemi ActiveTwo amplifier. EEG signals were continuously digitized and recorded at a sampling rate of 512 Hz, 24-bit A/D conversion. Left and right mastoids were selected as offline reference electrodes. Two active electrodes were attached below and above the left eye for a vertical electro-oculogram (VEOG) recording; likewise, two active electrodes were pasted at the orbital rim of both eyes for a horizontal electro-oculogram (HEOG) recording.

The BrainVision Analyzer 2 (Brain Products, Gilching, Germany) was chosen to process the collected EEG data offline. Re-referencing of signals was applied to the algebraic average of the two mastoid channels, which served as a reference. Next, EEG data was filtered with a 0.1–30 Hz bandpass filter as well as a 50 Hz notch filter. Subsequently, an ocular correction was implemented by the BrainVision Analyzer 2 using Gratton’s algorithm (Gratton et al., 1983). This was followed by EEG data of 200 ms before and 1,000 ms after the onset of employee presentations, and was segmented and baseline-corrected using the 200 ms time window prior to stimulus onset. Finally, bad epochs with large amplitudes (exceeding ±100 μV) were excluded, by applying the max-min criteria to all 34 channels on the scalp, which ensured that only artifact-free trials were analyzed in further analyses. Bad channels recognized by waveform observation were interpolated by spherical splines before artifact rejection, to avoid unnecessary rejection caused by individual bad channels. Participants with more than 20% of their data consisting of bad epochs were removed from the participant set because of their low signal-to-noise ratio.

### Statistical Analysis

Prior to evaluating the ERP data statistics, time windows and regions of interest (ROIs) were chosen according to previous studies and observations of grand averaged waveforms.

N2 and N400 were suggested to have a midline fronto-central scalp distribution (Kanske and Kotz, 2010; Amoruso et al., 2013). Therefore, the left cluster (F3, C3), midline cluster (Fz, FCz, and Cz) and right cluster (F4, C4) were selected as ROIs, and the average value of every cluster was calculated before going into further statistical analysis. For the LPP, the left cluster (CP1, P3, PO3, and O1), midline cluster (Cz, CPz, Pz, and Oz) and right cluster (CP2, P4, PO4, and O2) were chosen as ROIs based on the statement that the LPP was maximal at the centro-parietal region (Schupp et al., 2000, 2004a,b).

For the analyses of N2, a time window from 150 ms to 350 ms after the onset was selected according to previous literature (Johnson, 1989; Van Dillen and Derks, 2012) and the inspection of grand averaged waveforms. Consistent with previous research, the mean amplitude of N400 between 350 and 500 ms after the target stimuli onset was analyzed (Federmeier and Kutas, 2002; Painter and Koelsch, 2011). Lastly, the LPP during the 500 – 800 ms time window after stimulus onset was chosen for statistical analyses (Weymar et al., 2010; Gootjes et al., 2011; Langeslag and van Strien, 2013).

Repeated measures ANOVA (analysis of variance) were conducted in SPSS 25, and the Greenhouse–Geisser correction was used, when necessary. Further pairwise comparison results were adjusted by the Bonferroni correction to counteract the problem of multiple comparisons.

## Results

Results of reaction time suggested the successful manipulation in our task (see Supplementary Material). In evaluation stages, given the effects of context (either the main effect or interaction effect) during different time windows (see Supplementary Table 1), we analyzed data in the economic and social network contexts separately because: (a) the effects of context (either main effect or interaction effect) were observed in every stage; and (b) the goal in this manuscript is to investigate the decision-making process and the decision-making results in different contexts. Reporting results in different contexts can make the analysis part clearer and more readable. Following the evaluation processes, N2 (150–350 ms after onset), N400 (350–500 ms after onset), LPP (500–800 ms after onset), and self-reported feelings of comfort/discomfort (button press) were analyzed successively in the economic and social network contexts.

### Economic Context

In this section, only data in the economic context were analyzed. Three successive ERP components and self-reported data were assigned in within-subjects or repeated measures ANOVA. Note that region is the factor we are not interested in, and the main effect of region and the interaction effect of region will not be mentioned or discussed.

#### N2

A 2 (warmth: high vs. low) × 2 (competence: high vs. low) × 3 (region: right vs. middle vs. left) repeated measures ANOVA on the N2 component, revealed no main effects of warmth and competence or interaction effects between them (*p* > 0.05).

#### N400

**FIGURE 3:**
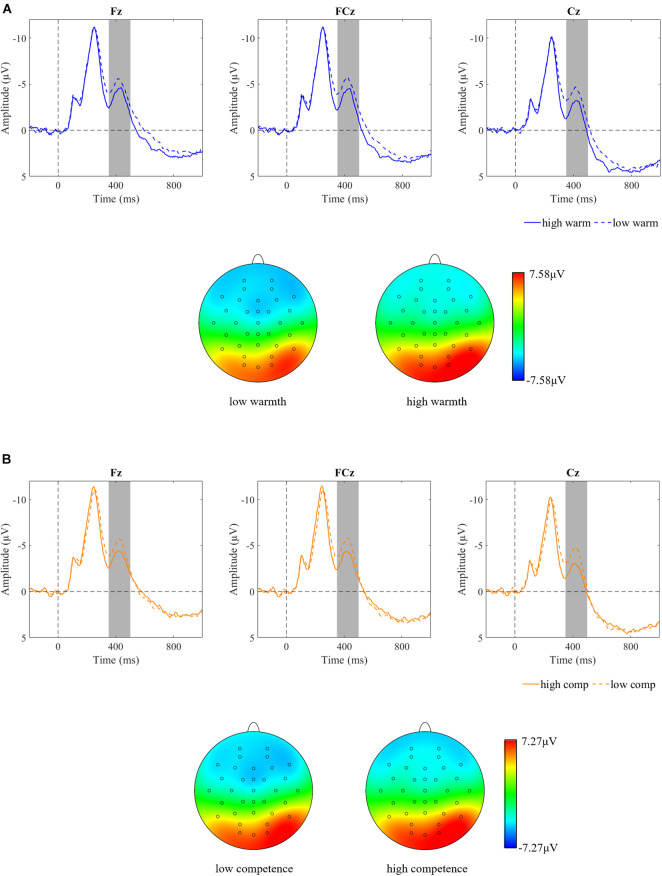
Brainwaves and scalp topographies of the N400 activity for two dimensions. **(A)** Warmth dimension. **(B)** Competence dimension.

**FIGURE 4:**
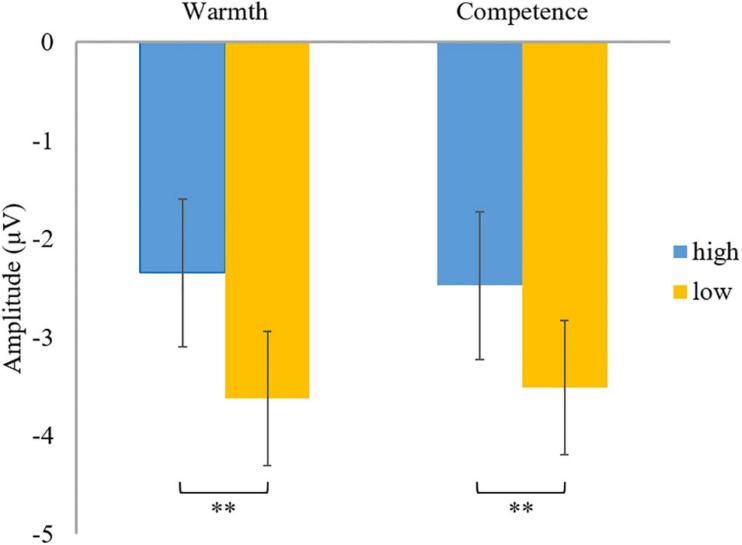
Descriptive statistics and contrasts for the N400 component (^∗∗^*p* ≤ 0.01).

A 2 (warmth: high vs. low) × 2 (competence: high vs. low) × 3 (region: right vs. middle vs. left) repeated measures ANOVA on N400, confirmed the main effects of warmth [*F*(1,44) = 11.43, *p* = 0.002, ηp^2^ = 0.21; Figure 3A] and competence [*F*(1,44) = 8.87, *p* = 0.005, ηp^2^ = 0.17; Figure 3B], but a non-significant interaction effect between them [*F*(1,44) = 3.17, *p* = 0.082, ηp^2^ = 0.067]. Subsequent pairwise comparisons (see Figure 4) showed more negative N400 values when facing employees with low warmth than those with high warmth. Similarly, low competence was proven to cause a larger N400 amplitude than high competence (see Figure 4). Consistent with our hypothesis, employees with high competence elicited larger N400 amplitudes than employees with low competence. However, a significant main effect of warmth on N400 was also observed. Therefore, in the economic context, our data partially supported the hypothesis of N400.

#### LPP

**FIGURE 5:**
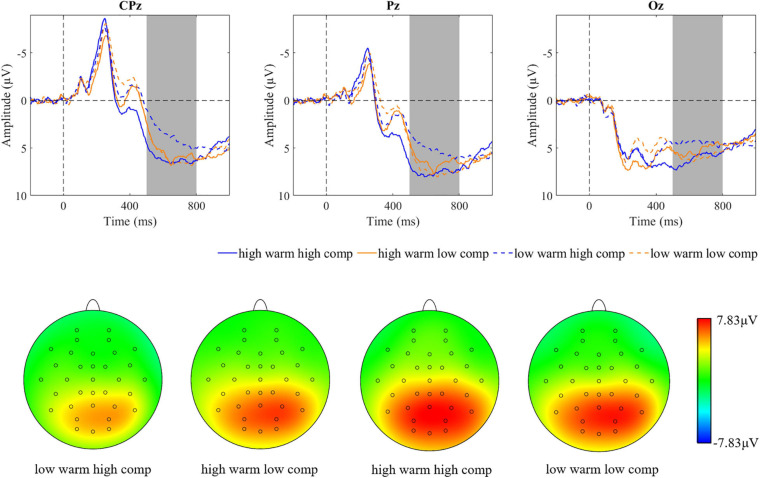
Brainwaves and scalp topographies of the late positive potential (LPP) activity for four categories.

**FIGURE 6:**
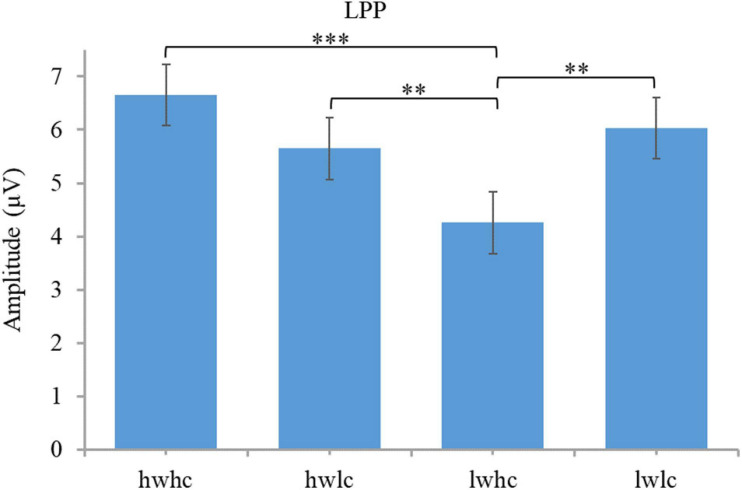
Descriptive statistics and contrasts for the LPP (hw, high warmth; lw, low warmth; hc, high competence; lc, low competence; ^∗∗^*p* ≤ 0.01 and ^∗∗∗^*p* ≤ 0.001).

A 2 (warmth: high vs. low) × 2 (competence: high vs. low) × 3 (region: right vs. middle vs. left) repeated measures ANOVA was conducted on the LPP. The results showed main effects of warmth [*F*(1,44) = 12.25, *p* = 0.001, ηp^2^ = 0.22], but not competence [*F*(1,44) = 1.23, *p* = 0.30, ηp^2^ = 0.025]. The interaction effect between warmth and competence reached a significant level [*F*(1,44) = 11.73, *p* = 0.001, ηp^2^ = 0.21; Figure 5]. Statistical results for the LPP by category are presented in Figure 6. A paired *t*-test of four SCM categories showed that a smaller LPP amplitude was evoked by cold but competent employees, than that evoked by employees in the other three categories. The results of the LPP partially supported the hypothesis because competence did not play a role in the LPP alone, but warmth and competence played a joint role in influencing the LPP amplitude.

#### Self-Reported Feelings

**FIGURE 7:**
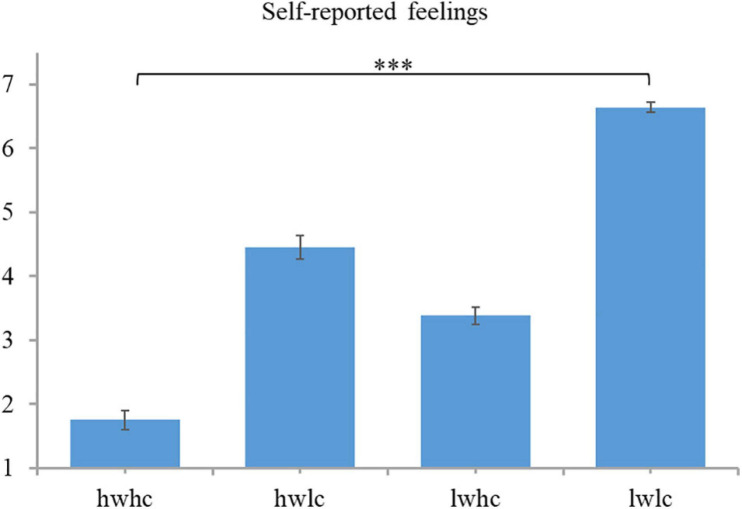
Descriptive statistics and contrasts for self-reported feelings (hw, high warmth; lw, low warmth; hc, high competence; lc, low competence; ^∗∗∗^*p* ≤ 0.001).

For self-reported feelings, a 2 (warmth: high vs. low) × 2 (competence: high vs. low) repeated measures ANOVA was conducted. A main effect of warmth [*F*(1,44) = 345.25, *p* < 0.001, ηp^2^ = 0.89] and competence [*F*(1,44) = 239.27, *p* < 0.001, ηp^2^ = 0.85] was noted. The interaction effect between warmth and competence was also observed to be significant [*F*(1,44) = 6.77, *p* = 0.013, ηp^2^ = 0.13]. Further paired *t*-test results (see Figure 7) revealed the highest score for firing cold and incompetent employees, followed by warm but incompetent employees and then cold but competent employees. Firing warm and competent employees induced the lowest score (*p* < 0.001 in all pairwise comparisons). Note that in our scale, a value of 1 means uncomfortable, 4 means neutral, and 7 means comfortable; so, the results showed that managers felt the most uncomfortable when they had to fire all-good (warm and competent) employees, followed by competent jerks (cold but competent). Firing lovable but inept (warm but incompetent) and all-bad (cold and incompetent) employees, did not make them uncomfortable. Thus, these findings supported the competence-influenced self-reported feelings of comfort/discomfort, as did warmth.

### Social Network Context

In this chapter, only data in the social network context were analyzed. Three successive ERP components and self-reported data were assigned in within-subjects or repeated measures ANOVA. Note that region is the factor we are not interested in, and the main effect of region and the interaction effect of region will not be mentioned or discussed.

#### N2

A 2 (warmth: high vs. low) × 2 (competence: high vs. low) × 3 (region: right vs. middle vs. left) repeated measures ANOVA on N2, revealed no main effects of warmth and competence nor interaction effects between them (*p* < 0.05).

#### N400

**FIGURE 8:**
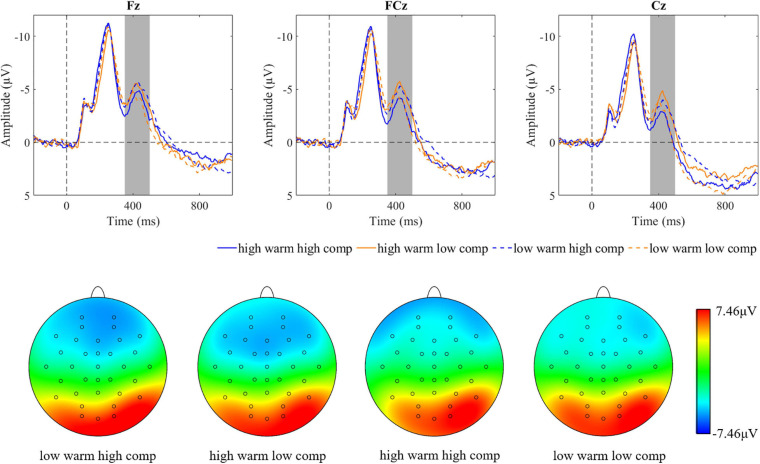
Brainwaves and scalp topographies of the N400 activity for four categories.

**FIGURE 9:**
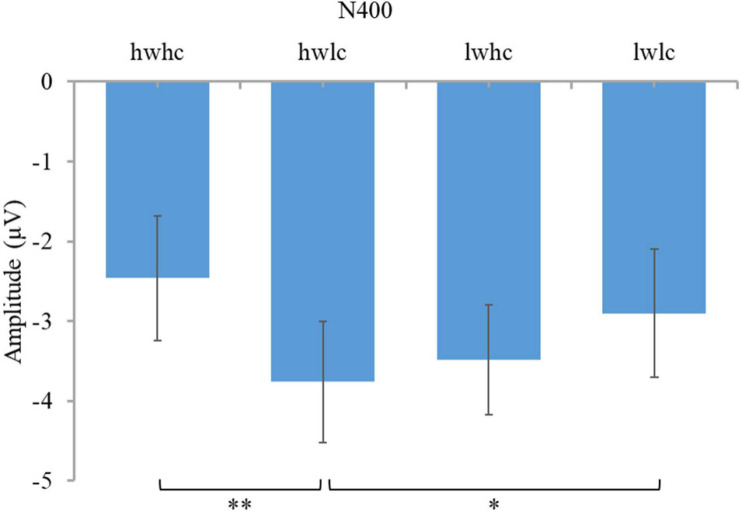
Descriptive statistics and contrasts for the N400 component (hw, high warmth; lw, low warmth; hc, high competence; lc, low competence; ^∗^*p* ≤ 0.05 and ^∗∗^*p* ≤ 0.01).

To understand the effect of warmth and competence on N400, a 2 (warmth: high vs. low) × 2 (competence: high vs. low) × 3 (region: right vs. middle vs. left) repeated measures ANOVA was conducted. The results suggested non-significant main effects of warmth [*F*(1,44) = 0.063, *p* = 0.80, ηp^2^ = 0.001] and competence [*F*(1,44) = 1.26, *p* = 0.27, ηp^2^ = 0.028]. However, a significant interaction effect of warmth and competence was observed [*F*(1,44) = 7.23, *p* = 0.010, ηp^2^ = 0.14; Figure 8]. Further paired *t*-tests (see Figure 9) revealed that the N400 amplitudes in two extreme groups (warm and competent, cold and incompetent) were smaller than those of the warm but incompetent group. Thus, the results indicated that warmth and competence interacted in influencing the N400 amplitude.

#### LPP

**FIGURE 10:**
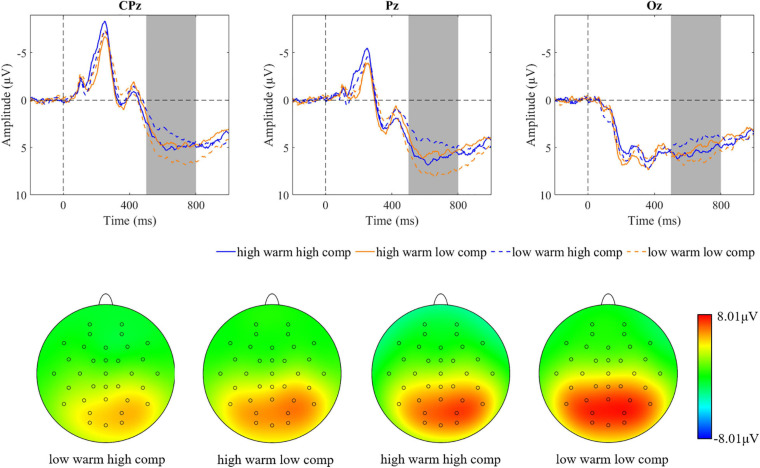
Brainwaves and scalp topographies of the LPP activity for four categories.

**FIGURE 11:**
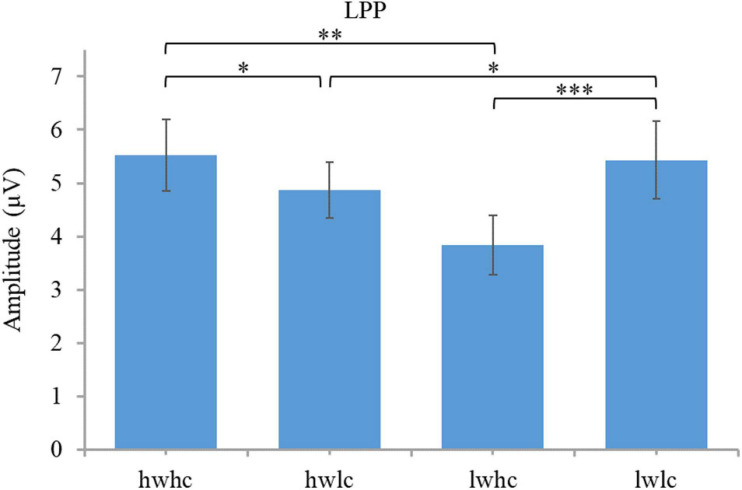
Descriptive statistics and contrasts for the LPP (hw, high warmth; lw, low warmth; hc, high competence; lc, low competence; ^∗^*p* ≤ 0.05, ^∗∗^*p* ≤ 0.01, and ^∗∗∗^*p* ≤ 0.001).

A 2 (warmth: high vs. low) × 2 (competence: high vs. low) × 3 (region: right vs. middle vs. left) repeated measures ANOVA was conducted on the LPP. The results revealed a significant main effect of competence [*F*(1,44) = 7.38, *p* = 0.009, ηp^2^ = 0.14] but not warmth [*F*(1,44) = 0.038, *p* = 0.85, ηp^2^ = 0.001]. In addition, the interaction effect of warmth and competence on the LPP was also proven to be significant [*F*(1,44) = 13.61, *p* = 0.001, ηp^2^ = 0.24; Figure 10]. Subsequent pairwise comparisons in Figure 11 showed that the two extreme groups (warm and competent, cold and incompetent) elicited larger LPP amplitudes than the two ambivalent groups (warm but incompetent, cold but competent) with all *p*-values < 0.05. The LPP findings supported the hypothesis.

#### Self-Reported Feelings

**FIGURE 12:**
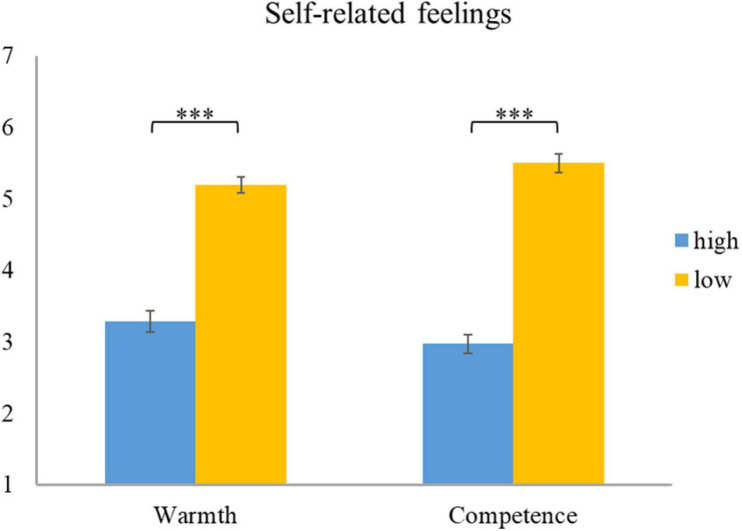
Descriptive statistics and contrasts for self-reported feelings (^∗∗∗^*p* ≤ 0.001).

A 2 (warmth: high vs. low) × 2 (competence: high vs. low) repeated measures ANOVA was conducted on self-reported discomfort data. The results showed significant main effects of warmth [*F*(1,44) = 108.11, *p* < 0.001, ηp^2^ = 0.71] and competence [*F*(1,44) = 213.17, *p* < 0.001, ηp^2^ = 0.83]. However, no interaction effect of the two factors was observed [*F*(1,44) = 053, *p* = 0.47, ηp^2^ = 0.012]. Subsequent pairwise comparisons revealed a lower score for high warmth compared to low warmth employees. Note that in our 1–7 scale, 1 represents uncomfortable while 7 represents comfortable. The results show that managers felt more uncomfortable when they had to socially exclude warm employees (see Figure 12). Similarly, the lower score for high competence and then low competence employees indicates greater discomfort, induced by excluding competent employees (see Figure 12). To summarize, consistent with our hypothesis, warmth and competence worked independently in influencing the self-reported feeling of comfort/discomfort when excluding employees. Specifically, excluding warm and excluding competent employees led to greater discomfort among managers.

## Discussion

This manuscript focused on how managers make decisions (or evaluations) about their employees and we tested two main accounts of the dual-process theory: the default-interventionist account (Kahneman and Frederick, 2002) and the parallel-competitive account (Lieberman, 2000). These two competing accounts have been in the spotlight for quite a while, but have mostly been discussed at the theoretical level and inspired many researchers in organization theory, to further discuss the theoretical and practical implications of the theory (Hodgkinson and Sadler-Smith, 2018). Yet, as Hodgkinson and Sadler-Smith (2018) note: It is time to explicate the neural substrates of managerial decision-making. Especially note that the default-interventionist account of the dual-processing theory has been a very popular metaphor under the name “iceberg principle” to describe how managers make decisions. Hence, it is frequently mentioned in many managerial courses both at an MBA or executive education level^1^.

We used the SCM to study how target employees, categorized according to the SCM, might moderate the dual processes, allowing us to shed a more detailed light onto the processes involved in the two dual-process models. While the SCM has already been studied in an organizational context (Cuddy et al., 2011) the underlying neural correlates (or neural signatures as Cuddy et al., 2008 call it), have thus far not been studied in an organizational context in which different decisions have to be made. So, we focused on two distinct organizational contexts, specifically in the economic and social network context because, within firms, managers frequently make such evaluations or decisions keeping these agentic vs. communal goals in mind (Bakan, 1966; Bass and Stogdill, 1990), and these goals affect managers’ evaluation processes both implicitly and explicitly (Dijksterhuis and Aarts, 2010). In addition, we sought to study the two evaluation processes using ERPs within three different time windows of interest (N2, N400, and LPP) and focused on the reported feelings of comfort/discomfort, having laid off or socially excluded target employees (all were members of one of the four SCM categories). Specifically, such a study (although with ecological validity limitations) could offer a glimpse into how managers’ minds operate as they develop their evaluations needed to make the aforementioned two decisions (microfoundations of organizations). Given the novelty of the study, it was difficult to make very concrete conjectures or hypotheses. We initially conjectured that the evaluation processes in the economic context as opposed to the social network context, would show different neural dynamics affected by different aspects or a configuration of SCM categories. This is logical, given that the goals of both evaluations/decisions triggered by these two organizational contexts are different. Indeed, we found that various aspects of the SCM were relevant for specific goals and elicited different amplitudes during different time windows, as well as during the self-reported feelings after having fired or excluded a target employee. Generally, we observed complex dynamics regarding the brain as well as the self-reported affect, which is explained below.

Before discussing the main findings of the economic and social network contexts, it is important to mention that neither the basic dimensions (warmth and competence) nor the SCM categories (as expected) affected the N2 amplitudes during the first time window, known for its detection of novelty. In social neuroscience — albeit a point of discussion — it has been observed by merely watching pictures of people passively, that people can gauge the trust or warmth of a person at a speed of approximately 100 ms (Willis and Todorov, 2006), but contradictory findings are presented by Williams and Themanson (2011) and Perrin and Garcıa-Larrea (2003). The main reason for this finding or non-different finding in our study about the N2 time window is that, the participants differed in novelty detection, regardless of the context. In the experiment, target employees were not watched passively by the (aspiring) managers, because their evaluation task was part of a significant decision, namely, firing or excluding target employees. Apparently the participants in the experiment needed more time (more than 300 ms) to make evaluations, in which SCM aspects would be relevant.

In the economic context during the second time window, N400, which is known to be involved in goal violation, showed different amplitudes in different dimensions. As hypothesized, the low performers, as opposed to the better performers, evoked a larger N400 amplitude, but so did the target employees with low levels of warmth as opposed to the high warmth group (which had not been hypothesized). This has two implications: Firstly, managers at this time window, which operated at the unconscious level, did not experience goal violation with target employees according to the four SCM categories, but experienced goal violation with target employees based on two basic dimensions of the SCM. Secondly, managers at this time window only considered on an unconscious level, whether a target employee is a good performer as opposed to a bad performer, and whether a target employee is a warm or cold person; hence, they are called the “Big Two” (Abele and Wojciszke, 2013; Yzerbyt, 2016).

During the subsequent LPP time window, which indicated levels of emotional arousal and which occurred at the conscious level, managers began to distinguish target employees according to the four SCM categories. Employees in three SCM categories elicited a higher emotional arousal (thus higher amplitude) than those in the other SCM category (low warmth but high-performing). Target employees with high warmth and high competence evoked a high arousal (or amplitude), which had not been hypothesized, but in hindsight, this is plausible as these should be considered as the most desired or admired group of people (employees in this case) categorized according to the SCM. However, the high warmth and low competence, as well as the low warmth and low competence employees, also evoked high LPP amplitudes, which had not been expected either. Hence, we could interpret that the high warmth (but low competence) group might evoke a higher level of emotional arousal, as a manager’s main social motivation is to be in the company of warm employees, but these employees with high levels of warmth might trigger compassion or concern (which is emotionally arousing), if they have to be fired when the company undergoes tough financial times. Likewise, the low warmth and low-performing employees evoked a high LPP amplitude, because the manager might be delighted that this group of employees is finally leaving the organization. Most interestingly, employees with low levels of warmth but high levels of competence elicited the lowest LPP amplitude or lowest emotional arousal, which means that managers were indifferent to them. Note that this observation is similar to a finding by Cuddy et al. (2011) that such employees, although excellent performers, leave the manager indifferent, which is why they are called competent jerks (Casciaro and Lobo, 2005).

Most importantly and partially conforming to the hypothesis is that the high warmth and high competence employees, cause the greatest uncomfortable feelings when fired, followed by the low warmth and high competence group. This is logical, as the goal of the decision is to save the company from financial disaster and to keep the better performers. The other two groups logically evoke less intensive feelings of discomfort when fired; those with low levels of warmth and low levels of performance as well as those with high levels of warmth and low levels of performance. Here, again, this is reasonable, as it might indicate that the company would benefit from getting rid of the low performers. In short, these findings on self-reported discomfort are compelling, given the economic context where financial goals drive the decision. Note that the competent jerks, on the one hand, leave managers indifferent (low amplitude at the LPP component), but we speculate that they will not be fired from the organization as they evoke a high level of discomfort upon being fired. Note how this finding differs from the findings of Cuddy et al. (2011), which suggested that this group is disadvantaged in the workplace (compared to employees exhibiting warmth or desirable social characteristics). However, because of their competence in this phase, they are favored in organizational positions, since competence is the key driver in the financial growth and development of the company.

In conclusion, the decision-making or evaluation in the economic context shows interesting neural and psychological dynamics. With regard to N400, both the warmth and competence dimensions of the SCM elicit differences in amplitude, thus scores shown on these dimensions are already relevant at the unconscious level for the manager, but at later stages of the evaluation—and more at the conscious level—the four SCM categories separately affect the amplitudes of the LPP and the intensity of the self-reported feelings. In short, this evaluation evolves from a coarse-grained evaluation in the unconscious phases to a more fine-grained evaluation in the conscious phases.

In the social network context, we proposed a combination of both economic and social goals, which means that employees need to be both similar to the manager as well as productive. For the N400 (reflecting goal violation), the observed amplitude differences reflect the four different SCM categories. More specifically, the target employees belonging to the high warmth and low competence group evoke the highest N400 amplitude. The other groups, namely, the low warmth and low-performing group, followed by the high warmth and high-performing group, and finally the low warmth and low performance group, evoked the lowest goal violation, thus eliciting the lowest N400 amplitude in this time window. We cannot logically explain this finding, as the low warmth and low performance group would normally evoke the highest amplitude. Again, in hindsight, it is apparent that the manager might feel the greatest conflict with the high warmth and low-performing group as they might be perceived as poor contributors to the social network, despite their similarity to the network members (or high warmth).

During the LPP time window, the way in which SCM categories affect the amplitudes of the LPP amplitude pattern, becomes more logical as they fit the social network goals. Firstly, the two extreme categories elicited higher LPP amplitudes compared to the two ambivalent categories. More specifically, the low warmth and low-performing group evoked high emotional arousal, similar to the response in the economic context, making the manager happy (or so we assume) to get rid of these employees from their social network. Similarly, the high warmth and high-performing group also evoked high arousal (and thus higher amplitude), as managers prefer this socially similar and productive group of employees in the company. The high warmth and low-performing group evoked low arousal (lower LPP amplitude), for which we have no direct explanation. However, similar to the economic context, the low warmth and low-performing group (competent jerks) evoked the lowest arousal (lowest amplitude), which, as explained, occurs because they generally leave people (colleagues) indifferent.

What is surprising in this social network context is that, when managers were asked what they feel about excluding groups of employees, they made coarse-grained evaluations; only the high warmth and the high-performing groups evoked discomfort, and this discomfort is lower (but not quite comfortable) for the low warmth and low-performing group, which is logical. In lay terms, managers prefer warm employees and better-performing employees and detest excluding them from their social network.

In short, when making evaluations related to the social network context, the early time windows that reflect mental processes at the unconscious level, already indicate that the SCM categories affect the amplitude of the early ERP components, and do so in the more conscious phase. However, at a later stage (reporting the degree of self-reported feelings upon having to exclude the employee), managers only focus on more coarse-grained dimensions of the SCM (warmth and competence).

All in all, different aspects of the SCM affect the ERP amplitudes during different time windows of interest and the self-reported feelings at a later stage. We now address how these findings about fine-grained time windows relate to the dual-processing accounts of evaluation mentioned at the beginning of the manuscript; the default-interventionist account, and the other is the parallel-competitive account (Hodgkinson and Sadler-Smith, 2018). On the assumption that we view our evaluation phases as part of two types of processes: Type 1 (which includes the N2 and the N400) vs. Type 2 (which includes the LPP and the self-reported evaluations); how would the results of this study fit these two dual-process perspectives?

Firstly, in accordance to the default-interventionist account, Type 1 decision processes are considered to be more unconscious, inflexible, holistic, automatic and evolutionarily old, whereas Type 2 decision processes are considered to be explicit, flexible, abstract and logical (Evans, 2008; Frith and Frith, 2008). More importantly, Type 2 decision processes cannot be or is difficult to be overruled by Type 1 decision processes. However, this description of the phases does not correspond with our findings. As this study shows, the evaluative processes show continuously dynamic changes during specific processes in different time windows or phases at the unconscious level (lasting from a few milliseconds to 400 ms). More specifically, in the social network context, fine-grained SCM-based distinctions occurred in early time windows, but in the economic contexts, early time windows reflected more coarse-grained SCM distinctions. This shows that these early time windows (or implicit processes as they are called), are not inflexible or holistic, as is typically assumed in the default-interventionist accounts (Nisbett et al., 2001; Frith and Frith, 2008). These early time windows show much flexibility in how aspects of SCM modulate amplitudes due to the goals that rule the context.

It is more likely that our data corresponds with the parallel-composite account of the dual model, which proposes that Type 1 and Type 2 processes are dynamic and recursive and that one might be more dominant in how the evaluation unfolds (Healey and Hodgkinson, 2014). Specifically, although the time windows were sequential by definition, aspects of the SCM modulated the elicited amplitudes in highly dynamic ways across all the time windows of interest, as did the self-reported feelings, because they were driven by the evaluation goals. Thus, Type 1 processes did not overrule Type 2 processes, but both were active simultaneously.

What could be the implication for managers? Managers are frequently taught that they should believe in their gut feelings, which means that they should rely on their intuitions (i.e., Type 1 processes). These intuitions reflect the implicit processes and they should learn to let these intuitions guide their more explicit or slow called “rational” deliberations (refer to Highhouse, 2008 for a good discussion).

Our study, however, shows the wide changes in dynamics across contexts that come with distinctive goals and the different evaluation phases that should inform managers of the dynamics. In other words, as the data shows, managers make many varying modifications in their evaluation processes, but Type 1 phases do not evoke inertia. So, when researchers or coaches train managers, it is important not to overemphasize the iceberg principle, which is a metaphor that reflects the default-interventionist account. The practical implication of this study is merely to teach managers to not only allow their gut feelings to guide them when making evaluations, but they should seek flexibility in their thinking (refer to Hodgkinson and Sadler-Smith, 2011 for a somewhat similar perspective).

Another implication of the study is that, although the competent jerks are not the most popular employees with regard to emotional arousal as they are conceived as cold, yet with regard to self-reported feelings when firing them, they suddenly evoke much discomfort as – in the economic context – managers do not want to fire them. In contrast, the results show that managers constantly operate with dissonant information and operate as ambidextrous learners (Hodgkinson and Healey, 2011).

Finally and in general, this study only focuses on one aspect of what is known as organizational neuroscience. Therefore, we do not pretend to have uncovered the holy grail of organizational neuroscience and have focused on one aspect of microfoundations of organizations. Yet, once again, we propose two main findings: (A) The dual-process model is not a productive guidance for research in organization theory and it is in need of refinement, as organizational context moderates the dynamics of this process; (B) Given that the so-called iceberg decision-making model (which corresponds to the default-interventionist account) is used in so many managerial courses, this study reflects a wake-up call for executive teachers or researchers in organization theory, who teach executives not to simplify the dynamics in managerial decision-making, but to accentuate how managerial decision-making implies complex dynamics given the context in which this decision-making takes place.

## Limitations and Suggestions for Future Research

This study is an exploratory study, yet which contains many validity checks such as using the validation study of the SCM stimuli, with a large sample of participants and using both physiological and self-reported measures to uncover the dynamics of managerial decision-making processes. Such a study, by definition, has limitations and is thus in need of replication. Hence, we invite researchers to replicate this study. Note too, given the relative newness and complexity of this study, we could not make concrete and very specific hypotheses in this manuscript, but limited ourselves to several plausible conjectures.

The following initially discusses the limitations of the study in relation to the design or technical aspects, and then broaches on limitations related to the sample being used. This allows us to propose some suggestions for future research.

Future research could expand this study by adding more time components (such as P300) as well as using different contexts, as they have different goals. Different aspects of social stimuli should play a role in the various (early and late) stages of ERP dynamics. In doing so, researchers can better understand how managers engage in evaluations when making decisions.

Based on the theories of picture superiority, images are easier to recall and recognize compared to words (Mcbride and Dosher, 2002). In addition, unlike words that might trigger the same ERPs to appear in different time windows because of the individual’s different reading speeds, image stimuli lead to fewer differences regarding the ERP time windows. However, picture stimuli might also contain some uncontrollable factors that might influence the results, such as facial expressions, gender, and the background of the pictures in our study. Despite the textual description of the warmth and competence of social targets, other factors should have been well controlled. This could be addressed in future studies.

Besides, in our study stimuli were manipulated to correspond with the SCM categories. However, Koch et al. (2016) argued that this manipulation constrains stereotypes into two dimensions. Without this constraint, people may spontaneously use other relevant dimensions. We hope researchers can also include other dimensions to extend our research.

As already suggested, an ERP experiment in a laboratory setting, by definition, is an artificial context and, for many reasons, does not mimic precisely how managers actually go about making decisions in daily practice. In the first instance, managerial decision-making takes place over longer periods of time and occurs most of the time after consultation with colleagues and teams (such as human resource managers, higher management, etc.). Therefore, an ERP study only provides a snapshot of how managers make decisions. A research question could be phrased as: “What if managers participate in the experiment twice and would such a design reveal different results?”

In the experiment we asked students who are aspiring to become managers to participate. It would be productive to conduct this experiment with managers who have several years of experience. A similar study could be conducted with experienced professionals and with managers who also work in different departments in organizations, such as human resource professionals, top managers, or line managers. The reason being, that both their position and experiences equip them with different perspectives or goals, which then affect the neural and psychological dynamics of decision-making processes. In addition, as industries matter (consider high-tech firms, fast-moving producers, or government agencies) these described dynamics in managerial decision processes, might differ substantially among managers who operate in these industries.

Moreover, although we used physiological and self-reported measures, it would be productive to combine laboratory experiments with actual field studies. For example, managers could be asked to express how they go about making managerial decisions in daily practice. Additionally, because managers make decisions in teams, we could study how the brain and psychological dynamics of a top manager compared to a human resource of the same firm and having similar years of experience, would unfold in a similar ERP experiment. Or we could select successful in comparison to less successful managers (e.g., high failure as managers in terms of organization or team successes) and study whether they show different dynamics in their decision-making.

## Data Availability Statement

The raw and processed datasets of the current study are available from the corresponding authors upon reasonable request.

## Ethics Statement

The studies involving human participants were reviewed and approved by Erasmus Institute for Research in Management (ERIM), Erasmus University Rotterdam. The patients/participants provided their written informed consent to participate in this study.

## Author Contributions

HS: conceptualization, data collection, and writing – original draft preparation. WV: conceptualization and writing – original draft preparation. FB: writing – review and editing. JS: methodology and data collection. LW: methodology, supervision, and writing – review and editing. All authors contributed to the article and approved the submitted version.

## Conflict of Interest

The authors declare that the research was conducted in the absence of any commercial or financial relationships that could be construed as a potential conflict of interest.

## Publisher’s Note

All claims expressed in this article are solely those of the authors and do not necessarily represent those of their affiliated organizations, or those of the publisher, the editors and the reviewers. Any product that may be evaluated in this article, or claim that may be made by its manufacturer, is not guaranteed or endorsed by the publisher.

## Footnotes

1
https://www.oxfordleadership.com/the-iceberg-principle/
