# Supplementary material for: Investigating Managers’ Fine-Grained Evaluation Processes in Organizations: Exploring Two Dual-Process Perspectives
Source: Front Neurosci. 2021 Sep 1;15:649941. doi: 10.3389/fnins.2021.649941 (PMC8445034; doi:10.3389/fnins.2021.649941)
Supplement: Supplementary file 1 [file Data_Sheet_1.docx]

Supplement Information

Manipulation Check

In this study, reaction time from the participants were applied for the manipulation check. Reaction time measured the duration from the question presented to button press action entered into a 2 (context: economic vs. social network) × 2 (warmth: high vs. low) × 2 (competence: high vs. low) repeated measures ANOVA. Neither the main effect of context nor the interaction effect including context was found to be significant (*p* > .05). This result showed that given different scenario descriptions, participants paid equally close attention to the two different contexts. Furthermore, a salient interaction effect between warmth and competence was observed (F (1.44) = 77.43, *p* < .001), and subsequent paired t-test results revealed that the reaction time for ambivalent categories was significantly longer than extreme categories (*p* < .001), indicating that participants perceived conflicts in warm but incompetent as well as cold but competent groups; thus, participants spent more time making decisions about these two ambivalent groups compared with the extreme groups (Evans et al., 2015). This finding is consistent with the extreme and ambivalent categories in the SCM (Fiske et al., 2002).

ANOVA analysis with all factors

A 3 (region: left vs. right vs. midline) × 2 (context: economic vs. social) × 2 (warmth: high vs. low) ×2 (competence: high vs. low) repeated measures ANOVA was utilized with the three ERP components (N2, N400, and LPP) and self-reported feeling evaluation. Region factor was omitted when analyzing self-reported discomfort measurements, leading to a three-factor repeated ANOVA of the self-reported data. Results in Table SI indicate the effects of context (either the main effect or interaction effect) at different decision-making stages.

Table 1. F and p-value for significant effects of context

| Measure | N2 (150-350 ms) | N400 (350-500 ms) | LPP (500-800 ms) | Feeling |
| --- | --- | --- | --- | --- |
| Main effect |  |  |  | **context** (F (1,44) = 4.35, *p* = .043) |
| Second order interaction | region × **context** (F (2, 88) = 3.66, *p* = .030) | **context** × warmth (F (1, 44) = 7.26, *p* = .010) | region × **context** (F (2, 88) = 5.67, *p* = .005)  **context** × warmth (F (1, 44) = 4.24, *p* = .046) | **context** × competence (F (1,44) = 7.68, *p* = .008) |
| Third order interaction |  |  | region × **context** × competence (F (2, 88) = 3.41, *p* = .038) |  |
| Forth order interaction |  |  |  |  |

References

Evans, A.M., Dillon, K.D., and Rand, D.G. (2015). Fast but not intuitive, slow but not reflective: Decision conflict drives reaction times in social dilemmas. *Journal of Experimental Psychology: General* 144**,** 951.

Fiske, S.T., Cuddy, A.J., Glick, P., and Xu, J. (2002). A Model of (Often Mixed) Stereotype Content: Competence and Warmth Respectively Follow From Perceived Status and Competition. *Journal of Personality and Social Psychology* 82**,** 878-902.
